# Supplementary material for: Modified WCRF/AICR Score and All-Cause, Digestive System, Cardiovascular, Cancer and Other-Cause-Related Mortality: A Competing Risk Analysis of Two Cohort Studies Conducted in Southern Italy
Source: Nutrients. 2021 Nov 10;13(11):4002. doi: 10.3390/nu13114002 (PMC8620807; doi:10.3390/nu13114002)
Supplement: Supplementary file 1 [file nutrients-13-04002-s001.zip › supplementary.tables.pdf]

Supplementary

Supplementary Table S1. Baseline Characteristics of Partecipants: MICOL/PANEL and NUTRIHEP Studies by Food Frequency Questionnaire. Castellana Grotte, Putignano (BA), Italy, 2005-2020

Variable	Food Frequency Questionnaire	
	YES	NO
N***	4.905 (93.0)	366 (7.0)
Age at Enrollment (years)*	51.42 (15.82)	45.56 (15.55)
DBP (mmHg)*	124.12 (17.82)	122.56 (16.27)
SBP (mmHg)*	76.76 (9.62)	77.76 (9.56)
Weight (kg)*	73.06 (14.97)	72.16 (16.83)
BMI (kg/m^2)*	27.51 (5.15)	26.59 (5.54)
Triglycerides (mmol/L)*	1.38 (1.02)	1.27 (0.95)
Total Cholesterol (mmol/L)*	5.10 (1.02)	4.98 (0.96)
HDL (mmol/L)*	1.33 (0.35)	1.34 (0.34)
LDL (mmol/L)*	3.14 (0.88)	3.05 (0.88)
GGT (µkat/L)*	0.25 (0.25)	0.24 (0.22)
Glucose (mmol/L)*	5.88 (1.40)	5.87 (1.53)
GPT (µkat/L)*	0.28 (0.22)	0.26 (0.19)
Sex***		
Female	2372 (92.6)	188 (7.4)
Male	2533 (93.4)	178 (6.6)
Age classes***		
≤30	422 (86.1)	68 (13.9)
31-35	457 (91.4)	43 (8.6)
36-40	585 (93.3)	42 (6.7)
41-45	518 (90.6)	54 (9.4)
46-50	444 (94.1)	28 (5.9)
51-55	478 (91.4)	45 (8.6)
56-60	537 (95.2)	27 (4.8)
61-65	416 (96.7)	14 (3.3)
66-70	370 (95.9)	16 (4.1)
71-75	325 (96.4)	12 (3.6)
>75	353 (95.4)	17 (4.6)
Smoke***		
Never/Former	4043 (95.1)	210 (4.9)
Current	839 (91.9)	74 (8.1)
Without Information	23 (21.9)	82 (78.1)
Education***		
Primary School	1350 (97.1)	41 (2.9)
Secondary School	1483 (94.8)	82 (5.2)
High School	1391 (94.2)	86 (5.8)
Graduated	497 (95.0)	26 (5.0)
Illiterate	170 (98.3)	3 (1.7)
Without Information	14 (9.9)	128 (90.1)
Job***		
Managers & Professionals	287 (94.4)	17 (5.6)
Craft. Agricultural and Sales Workers	1179 (93.6)	81 (6.4)
Elementary Occupations	1132 (95.3)	56 (4.7)
Housewife	634 (96.5)	23 (3.5)
Pensioneers	1371 (97.4)	36 (2.6)
Jobless	254 (91.0)	25 (9.0)
Without Information	48 (27.3)	128 (72.7)
Marital Status***		
Single	765 (94.6)	44 (5.4)
Married/Coupled	3667 (96.6)	129 (3.4)
Separated/Divorced	117 (97.5)	3 (2.5)
Widower	314 (98.4)	5 (1.6)

Without information	42 (18.5)	185 (81.5)

SBP: Systolic Blood Pressure; DBP: Diastolic Blood Pressure; BMI: Body Mass Index; GGT: γ-Glutamyltransferase; GPT:Glutamate Pyruvate Transaminase HDL: High Density Lipoprotein Chiolesterol; LDL: Low Density Lipoprotein Cholesterol;; Cells reporting subject characteristics contain * Mean ±(SD). *** Number (Percentage)

Supplementary Table S2. Characteristics of Participants by modified WCRF/AICR Score Categories MICOL/PANEL Studies. Castellana Grotte (BA), Italy, 2005-2020

	All ¥	Modified WCRF/AICR Score Categories¥		
	0-11¥	≤5	5.5-7	>7
N	2848	576	1815	457
Age at Enrollment (years)*	54.70 (14.95)	51.37 (13.33)	54.13 (14.73)	61.18 (15.81)
DBP (mmHg)*	123.45 (19.80)	122.99 (17.49)	122.98 (19.85)	125.91 (22.11)
SBP (mmHg)*	74.69 (10.16)	76.59 (10.67)	74.42 (10.01)	73.38 (9.76)
Weight (kg)*	75.07 (15.26)	83.43 (14.76)	74.58 (14.81)	66.46 (11.94)
BMI (kg/m^2)*	28.65 (5.25)	30.36 (4.97)	28.54 (5.31)	26.95 (4.66)
Kcal days	2268.16 (836.65)	2770.11 (1.006.47)	2223.29 (732.33)	1794.05 (629.41)
Triglycerides (mmol/L)*	1.45 (1.02)	1.60 (1.12)	1.44 (1.03)	1.30 (0.76)
Total Cholesterol (mmol/L)*	5.16 (1.00)	5.25 (0.97)	5.15 (1.01)	5.07 (0.99)
HDL (mmol/L)*	1.32 (0.36)	1.26 (0.33)	1.32 (0.37)	1.40 (0.37)
LDL (mmol/L)*	3.17 (0.86)	3.26 (0.83)	3.16 (0.86)	3.07 (0.88)
Glucose (mmol/L)*	6.05 (1.51)	6.15 (1.32)	6.01 (1.45)	6.11 (1.87)
GPT (μkat/L)*	0.30 (0.25)	0.33 (0.23)	0.28 (0.22)	0.31 (0.37)
GGT(μkat/L)*	0.28 (0.29)	0.33 (0.33)	0.26 (0.26)	0.26 (0.34)
Sex***				
Female	1226 (43.0)	102 (17.7)	812 (44.7)	312 (68.3)
Male	1622 (57.0)	474 (82.3)	1003 (55.3)	145 (31.7)
Age (categorical. years)				
<40	621 (21.8)	138 (24.0)	418 (23.0)	65 (14.2)
40-49	588 (20.6)	148 (25.7)	378 (20.8)	62 (13.6)
50-59	560 (19.7)	141 (24.5)	352 (19.4)	67 (14.7)
60-69	525 (18.4)	86 (14.9)	345 (19.0)	94 (20.6)
≥70	554 (19.5)	63 (10.9)	322 (17.7)	169 (37.0)
Smoke***				
Never/Former	2319 (81.4)	452 (78.5)	1475 (81.3)	392 (85.8)
Current	529 (18.6)	124 (21.5)	340 (18.7)	65 (14.2)
Observation time**	14.95 (14.12. 15.33)	14.95 (14.80. 15.30)	14.95 (14.12. 15.33)	14.95 (12.94. 15.34)
Age at Death (years)*	69.24 (56.08. 79.95)	64.49 (55.07. 74.50)	68.63 (55.55. 79.28)	77.05 (61.93. 86.04)
Status***				
Alive and/or Censored	2274 (79.8)	491 (85.2)	1469 (80.9)	314 (68.7)
Dead	574 (20.2)	85 (14.8)	346 (19.1)	143 (31.3)
Cause of Death***				
Alive and/or Censored	2272 (79.8)	490 (85.1)	1468 (80.9)	314 (68.7)
DSD-related mortality	104 (3.7)	20 (3.5)	57 (3.1)	27 (5.9)
CVD-related mortality	161 (5.7)	22 (3.8)	101 (5.6)	38 (8.3)
CR-related mortality	97 (3.4)	18 (3.1)	59 (3.3)	20 (4.4)
Other-Cause mortality	214 (7.5)	26 (4.5)	130 (7.2)	58 (12.7)
Education***				
Primary School	847 (29.7)	159 (27.6)	535 (29.5)	153 (33.5)
Secondary School	888 (31.2)	188 (32.6)	556 (30.6)	144 (31.5)
High School	685 (24.1)	150 (26.0)	445 (24.5)	90 (19.7)
Graduated	324 (11.4)	56 (9.7)	217 (12.0)	51 (11.2)
Illiterate	104 (3.7)	23 (4.0)	62 (3.4)	19 (4.2)
Job***				
Managers & Professionals Craft. Agricultural and Sales Workers	125 (4.4)	35 (6.1)	75 (4.1)	15 (3.3)
Elementary Occupations	716 (25.1)	169 (29.3)	460 (25.3)	87 (19.0)
Housewife	701 (24.6)	147 (25.5)	464 (25.6)	90 (19.7)
Pensioneers	322 (11.3)	40 (6.9)	219 (12.1)	63 (13.8)
Jobless	938 (32.9)	176 (30.6)	571 (31.5)	191 (41.8)
Marital Status***				
Single	309 (10.8)	58 (10.1)	200 (11.0)	51 (11.2)

Married/Coupled	2208 (77.5)	477 (82.8)	1424 (78.5)	307 (67.2)
Separated/Divorced	84 (2.9)	17 (3.0)	52 (2.9)	15 (3.3)
Widower	247 (8.7)	24 (4.2)	139 (7.7)	84 (18.4)
Diabetes***				
No	2569 (90.2)	529 (91.8)	1650 (90.9)	390 (85.3)
Yes	279 (9.8)	47 (8.2)	165 (9.1)	67 (14.7)
Dyslipidemia ***				
No	2120 (74.4)	430 (74.7)	1365 (75.2)	325 (71.1)
Yes	728 (25.6)	146 (25.3)	450 (24.8)	132 (28.9)
Hypertension***				
No	1895 (66.5)	406 (70.5)	1220 (67.2)	269 (58.9)
Yes	953 (33.5)	170 (29.5)	595 (32.8)	188 (41.1)

GGT: γ-Glutamyltransferase ; GPT:Glutamate Pyruvate; BMI: Body Mass Index; DBP: Diastolic Blood Pressure; SBP: Systolic Blood Pressure; HDL: High Density Lipoprotein Cholesterol; LDL: Low Density Lipoprotein Cholesterol; RChol: Remnant Cholesterol; CVD-related mortality: Cardiovascular Disease related mortality; DSD-related mortality: Digestive System Disease -related mortality. Cells reporting subject characteristics contain * Mean ±(SD). **Median (IQR). ***Number. ¥ (Percentage) Percentages calculated per column.

Supplementary Table S3. Characteristics of Participants by modified WCRF/AICR Score Categories NUTRIHEP Study, Putignano (BA), Italy, 2005-2020

	All [¥]	Modified WCRF/AICR Score Categories[¥]		
	0-11	≤5	5.5-7	>7
N	2018	195	1134	689
Age at Enrollment (years)*	46.88 (15.86)	41.58 (14.18)	45.78 (15.91)	50.17 (15.59)
DBP (mmHg)*	125.06 (14.33)	125.41 (13.84)	124.88 (14.27)	125.25 (14.59)
SBP (mmHg)*	79.80 (8.19)	80.32 (7.67)	79.63 (8.30)	79.93 (8.15)
Weight (kg)*	70.21 (14.07)	78.26 (15.77)	71.95 (14.18)	65.08 (11.38)
BMI (kg/m^2)*	25.90 (4.54)	27.17 (4.98)	26.24 (4.76)	24.99 (3.83)
Kcal days	2066.12 (794.82)	3054.06 (947.87)	2164.47 (699.66)	1624.63 (552.07)
Triglycerides (mmol/L)*	1.27 (0.91)	1.37 (1.13)	1.31 (0.90)	1.18 (0.84)
Total Cholesterol (mmol/L)*	5.03 (1.03)	4.98 (1.07)	4.98 (0.98)	5.11 (1.09)
HDL (mmol/L)*	1.35 (0.34)	1.24 (0.29)	1.33 (0.34)	1.42 (0.34)
LDL (mmol/L)*	3.11 (0.89)	3.10 (0.88)	3.08 (0.86)	3.17 (0.94)
Glucose (mmol/L)*	5.62 (1.19)	5.66 (0.97)	5.60 (1.04)	5.65 (1.46)
GPT (µkat/L)*	0.26 (0.17)	0.32 (0.18)	0.27 (0.19)	0.23 (0.11)
GGT(µkat/L)*	0.22 (0.18)	0.27 (0.18)	0.23 (0.20)	0.19 (0.14)
Sex***				
Female	1126 (55.8)	45 (23.1)	557 (49.1)	524 (76.1)
Male	892 (44.2)	150 (76.9)	577 (50.9)	165 (23.9)
Age (categorical, years)				
<40	735 (36.4)	89 (45.6)	451 (39.8)	195 (28.3)
40-49	403 (20.0)	51 (26.2)	214 (18.9)	138 (20.0)
50-59	456 (22.6)	35 (17.9)	256 (22.6)	165 (23.9)
60-69	259 (12.8)	10 (5.1)	130 (11.5)	119 (17.3)
≥70	165 (8.2)	10 (5.1)	83 (7.3)	72 (10.4)
Smoke***				
Never/Former	1710 (84.7)	151 (77.4)	950 (83.8)	609 (88.4)
Current	308 (15.3)	44 (22.6)	184 (16.2)	80 (11.6)
Observation time**	14.74 (14.21. 14.87)	14.74 (14.20. 14.86)	14.74 (14.21. 14.88)	14.75 (14.21. 14.87)
Age at Death (years)*	61.12 (48.68. 72.74)	55.63 (43.82. 64.98)	59.48 (47.47. 72.17)	65.29 (52.54. 75.61)
Status***				
Alive and/or Censored	1860 (92.2)	185 (94.9)	1044 (92.1)	631 (91.6)
Dead	158 (7.8)	10 (5.1)	90 (7.9)	58 (8.4)
Cause of Death***				
Alive and/or Censored	1860 (92.2)	185 (94.9)	1044 (92.1)	631 (91.6)
DSD-related mortality	27 (1.3)	0 (0.0)	19 (1.7)	8 (1.2)
CVD-related mortality	49 (2.4)	3 (1.5)	25 (2.2)	21 (3.0)
CR-related mortality	31 (1.5)	3 (1.5)	18 (1.6)	10 (1.5)
Other-Cause mortality	51 (2.5)	4 (2.1)	28 (2.5)	19 (2.8)
Education***				
Primary School	485 (24.0)	27 (13.8)	272 (24.0)	186 (27.0)
Secondary School	599 (29.7)	76 (39.0)	337 (29.7)	186 (27.0)
High School	700 (34.7)	74 (37.9)	388 (34.2)	238 (34.5)
Graduated	169 (8.4)	14 (7.2)	104 (9.2)	51 (7.4)
Illiterate	65 (3.2)	4 (2.1)	33 (2.9)	28 (4.1)
Job***				
Managers & Professionals	162 (8.0)	28 (14.4)	101 (8.9)	33 (4.8)
Craft. Agricultural and Sales Workers	563 (27.9)	58 (29.7)	305 (26.9)	200 (29.0)
Elementary Occupations	337 (16.7)	52 (26.7)	207 (18.3)	78 (11.3)
Housewife	312 (15.5)	15 (7.7)	142 (12.5)	155 (22.5)
Pensioneers	434 (21.5)	20 (10.3)	244 (21.5)	170 (24.7)
Jobless	208 (10.3)	22 (11.3)	134 (11.8)	52 (7.5)

Without Information	2 (0.1)	0 (0.0)	1 (0.1)	1 (0.1)
Marital Status***				
Single	455 (22.5)	56 (28.7)	279 (24.6)	120 (17.4)
Married/Coupled	1465 (72.6)	132 (67.7)	807 (71.2)	526 (76.3)
Separated/Divorced	33 (1.6)	2 (1.0)	16 (1.4)	15 (2.2)
Widower	65 (3.2)	5 (2.6)	32 (2.8)	28 (4.1)
Diabetes***				
No	1973 (97.8)	189 (96.9)	1113 (98.1)	671 (97.4)
Yes	45 (2.2)	6 (3.1)	21 (1.9)	18 (2.6)
Dyslipidemia ***				
No	1939 (96.1)	189 (96.9)	1095 (96.6)	655 (95.1)
Yes	79 (3.9)	6 (3.1)	39 (3.4)	34 (4.9)
Hypertension***				
No	1767 (87.6)	175 (89.7)	1004 (88.5)	588 (85.3)
Yes	251 (12.4)	20 (10.3)	130 (11.5)	101 (14.7)

GGT: γ-Glutamyltransferase ; GPT:Glutamate Pyruvate; BMI: Body Mass Index; DBP: Diastolic Blood Pressure; SBP: Systolic Blood Pressure; HDL: High Density Lipoprotein Cholesterol; LDL: Low Density Lipoprotein Cholesterol; RChol: Remnant Cholesterol; CVD-related mortality: Cardiovascular Disease related mortality; DSD-related mortality: Digestive System Disease -related mortality. Cells reporting subject characteristics contain * Mean ±(SD). ** Median (IQR). *** Number. ¥ (Percentage) Percentages calculated per column.

Supplementary Table S4. Sensitivity analysis to obtain age at death range. All causes mortality MICOL/PANEL and NUTRIHEP studies. Castellana Grotte, Putignano (BA), Italy,2005 - 2020.

Modified WCRF/AICR score >7		All Causes mortality			
Age at death (range)		N failures tot (N.failures in category>7)	HR	95% CI	AIC-BIC
≤90					
	Whole	622 (154)	0.75	0.57-1.00	**89-225**
	Female	232 (92)	1.38	0.75-2.52	**100-214**
	Male	390 (62)	0.56*	0.39-0.82	**10-125**
≤89					
	Whole	593 (140)	0.82	0.61-1.10	137-272
	Female	218 (85)	1.51	0.82-2.78	111-225
	Male	375 (55)	0.63*	0.43-0.92	18-164
≤88					
	Whole	563 (130)	0.88	0.65-1.19	167-302
	Female	207 (82)	1.57	0.81-3.03	106-220
	Male	356 (48)	0.71	0.48-1.05	85-200
≤87					
	Whole	533 (124)	0.87	0.64-1.18	182-317
	Female	201 (78)	1.70	0.87-3.32	62-175
	Male	332 (46)	0.67	0.45-1.01	131-246
≤86					
	Whole	503 (117)	0.90	0.65-1.24	198-332
	Female	191 (75)	1.59	0.74-3.40	57-170
	Male	312 (42)	0.74	0.49-1.11	147-262
≤85					
	Whole	469 (107)	0.93	0.67-1.29	223-357
	Female	179 (69)	1.70	0.75-3.84	61-173
	Male	290 (38)	0.75	0.49-1.15	171-285

Adjusted for: Hypertension , Glutamate Pyruvate Transaminase (µkat/L), γ-Glutamyltransferase µkat/L), Glucose (mmol/L), Triglycerides in range vs non in range Marital Status; Education, Smoke (Never/Former vs Current) and BMI: Body Mass Index (kg/m2)
*p< 0.05; HR: Hazard Ratio; AIC and BIC: Akaike's and Schwarz's Bayesian information criteria

Supplementary Table S5. Sensitivity analysis to obtain age at death range by cause-specific mortality. MICOL/PANEL and NUTRIHEP studies. Castellana Grotte, Putignano (BA), Italy, 2005 - 2020

Modified WCRF/AICR score >7					
Age at death (range)		N failures tot (N.failures in category>7)	SHR	95% CI	AIC-BIC
**DSD-Related Mortality**					
≤90					
	Whole	119 (30)	0.72	0.38-1.37	**1603-1713**
	Female	41 (21)	2.62	0.62-11.00	**484-575**
	Male	78 (9)	0.38*	0.15-0.97	**962-1054**
≤89					
	Whole	115 (27)	0.68	0.35-1.33	1550-1659
	Female	41 (21)	2.64	0.65-10.63	477-568
	Male	74 (6)	0.28*	0.09-0.92	916-1009
≤88					
	Whole	114 (27)	0.81	0.43-1.52	1501-1610
	Female	40 (21)	2.49	0.62-10.05	462-553
	Male	74 (6)	0.41	0.15-1.07	895-987
≤87					
	Whole	104 (26)	0.96	0.51-1.81	1370-1479
	Female	39 (20)	2.73	0.69-10.73	431-522
	Male	65 (6)	0.45	0.17-1.21	799-891
≤86					
	Whole	103 (26)	0.94	0.50-1.78	1331-1439
	Female	39 (20)	2.56	0.65-10.00	423-514
	Male	64 (6)	0.47	0.18-1.24	770-862
≤85					
	Whole	94 (23)	1.14	0.57-2.28	1223-1332
	Female	36 (18)	3.09	0.57-16.72	393-483
	Male	58 (5)	0.58	0.20-1.65	702-794
**CVD-related mortality**					
≤90					
	Whole	171 (46)	1.19	0.69-2.07	**2218-2327**
	Female	60 (27)	4.14	0.61-28.29	**671-762**
	Male	111 (19)	1.01	0.51-1.98	**1328-1421**
≤89					
	Whole	163 (43)	1.22	0.70-2.14	2093-2203
	Female	56 (26)	4.60	0.69-30.72	616-707
	Male	107 (17)	0.98	0.49-1.97	1271-1363
≤88					
	Whole	150 (37)	1.14	0.63-2.04	1928-2037
	Female	51 (24)	3.56	0.59-21.62	568-659
	Male	99 (13)	0.86	0.40-1.84	1177-1269
≤87					
	Whole	139 (36)	1.14	0.62-2.07	1779-1888
	Female	49 (23)	3.83	0.69-21.21	538-629
	Male	90 (13)	0.90	0.41-1.95	1071-1163
≤86					
	Whole	127 (33)	1.14	0.61-2.13	1627-1736
	Female	45 (21)	NS		
	Male	82 (12)	0.93	0.43-2.04	978-1070
≤85					
	Whole	118 (30)	1.16	0.62-2.17	1483-1591
	Female	41 (19)	NS		
	Male	77 (11)	0.84	0.38-1.86	900-991
**Cancer related mortality**					
≤90					

	Whole	124 (29)	0.63	0.35-1.12	**1766-1876**
	Female	52 (18)	1.90	0.42-8.60	**671-762**
	Male	72 (11)	0.43*	0.19-0.97	**932-1024**
≤89					
	Whole	119 (28)	0.69	0.38-1.25	1707-1916
	Female	50 (17)	1.95	0.43-8.88	650-741
	Male	69 (11)	0.50	0.22-1.12	900-992
≤88					
	Whole	118 (27)	0.68	0.37-1.25	1679-1789
	Female	50 (17)	1.83	0.40-8.43	640-731
	Male	68 (10)	0.48	0.21-1.11	882-974
≤87					
	Whole	116 (26)	0.67	0.36-1.22	1639-1748
	Female	49 (16)	1.83	0.40-8.41	625-716
	Male	67 (10)	0.49	0.21-1.12	858-950
≤86					
	Whole	112 (25)	0.72	0.38-1.37	1585-1693
	Female	47 (16)	3.08	0.35-27.09	600-691
	Male	65 (9)	0.49	0.20-1.16	834-925
≤85					
	Whole	111 (25)	0.74	0.39-1.40	1544-1653
	Female	47 (16)	3.23	0.35-30.10	587-678
	Male	64 (9)	0.50	0.21-1.18	808-899
**Other-Cause mortality**					
≤90					
	Whole	208 (49)	0.87	0.53-1.45	**2676-2785**
	Female	79 (26)	0.43*	0.21-0.89	**863-954**
	Male	129 (23)	1.12	0.58-2.15	**1536-1629**
≤89					
	Whole	196 (42)	0.83	0.50-1.39	2514-2623
	Female	71 (21)	0.40*	0.20-0.83	779-870
	Male	125 (21)	1.12	0.58-2.17	1480-1573
≤88					
	Whole	181 (39)	0.95	0.55-1.65	2339-2448
	Female	66 (20)	0.50	0.22-1.16	731-822
	Male	115 (19)	1.31	0.66-2.61	1373-1465
≤87					
	Whole	174 (36)	0.84	0.48-1.46	2219-2328
	Female	64 (19)	0.49	0.21-1.16	698-789
	Male	110 (17)	1.08	0.53-2.20	1297-1389
≤86					
	Whole	161 (33)	0.89	0.50-1.59	2052-2161
	Female	60 (18)	0.43*	0.19-0.96	650-741
	Male	101 (15)	1.30	0.66-2.57	1191-1282
≤85					
	Whole	146 (29)	0.83	0.45-1.53	1858-1967
	Female	55 (16)	0.49	0.20-1.22	588-678
	Male	91 (13)	1.22	0.59-2.54	1079-1170

Adjusted for: Hypertension , Glutamate Pyruvate Transaminase (μkat/L), γ-Glutamyltransferase μkat/L), Glucose (mmol/L), Triglycerides in range vs non in range Marital Status; Education, Smoke (Never/Former vs Current) and BMI: Body Mass Index (kg/m2)

DSD-related mortality: Digestive System Disease –related mortality, CVD-related mortality Cardiovascular Disease related mortality. AIC and BIC: Akaike's and Schwarz's Bayesian information criteria. NS: Not estimable. *p<0.05.

Supplementary Table S6. Mortality Rates (MR) and 95% Confidence Intervals (95% CI) for All Causes mortality, for DSD-related mortality, CVD-related, Cancer-related mortality and Other-Causes Mortality from modified WCRF/AICR MICOL/PANEL and NUTRIHEP studies. Castellana Grotte, Putignano (BA), Italy,2005 - 2020

	Modified WCRF/AICR score									WCRF/AICR continuos		
	≤5			5.5-7			>7					
	ND	MR	95% CI	ND	MR	95% CI	ND	MR	95% CI	ND	MR	95% CI
**All causes mortality**												
Whole Sample	88	1.79	1.45;2.21	380	1.98	1.79;2.19	154	1.97	1.68;2.31	622	1.95	1.80;2.11
Female	13	1.37	0.79;2.36	127	1.44	1.21;1.72	92	1.64	1.34;2.02	232	1.51	1.33;1.72
Male	75	1.89	1.51;2.38	253	2.44	2.15;2.76	62	2.80	2.18;3.59	390	2.36	2.13;2.60
**DSD-related mortality**												
Whole Sample	19	0.39	0.25;0.61	70	0.37	0.29;0.46	30	0.38	0.27;0.55	119	0.37	0.31;0.14
Female	2	0.21	0.05;0.84	18	0.20	0.13;0.32	21	0.37	0.24;0.57	41	0.27	0.20;0.36
Male	17	0.43	0.27;0.70	52	0.50	0.38;0.66	9	0.41	0.21;0.78	78	0.47	0.38
**CVD-related mortality**												
Whole Sample	22	0.45	0.30;0.69	103	0.54	0.44;0.65	46	0.59	0.44;0.79	171	0.54	0.46;0.62
Female	1	0.10	0.01;0.75	32	0.36	0.26;0.51	27	0.48	0.33;0.70	60	0.39	0.30;0.50
Male	21	0.54	0.35;0.82	71	0.69	0.55;0.87	19	0.85	0.55;1.34	111	0.68	0.56;0.81
**Cancer-related mortality**												
Whole Sample	21	0.43	0.28;0.66	74	0.38	0.30;0.48	29	0.37	0.26;0.53	124	0.39	0.32;0.46
Female	2	0.21	0.05;0.84	32	0.35	0.25;0.50	18	0.32	0.20;0.51	52	0.33	0.25;0.44
Male	19	0.49	0.31;0.76	42	0.41	0.30;0.55	11	0.50	0.27;0.90	72	0.44	0.35;0.55
**Other-Cause mortality**												
Whole Sample	26	0.51	0.35;0.76	133	0.69	0.58;0.82	49	0.63	0.47;0.83	208	0.65	0.56;0.74
Female	8	0.84	0.42;1.68	45	0.52	0.39;0.70	26	0.46	0.32;0.68	79	0.52	0.42;0.65
Male	18	0.43	0.27;0.70	88	0.83	0.68;1.03	23	1.04	0.69;1.556	129	0.77	0.64;0.91

DSD-related mortality: Digestive System Disease Deaths; CVD-related mortality Cardiovascular Disease Deaths, **Cancer-related mortality:** Cancer Deaths; Other-Cause mortality: Other Causes Deaths. MR: Mortality Rates for 1000 person-years ND: Number Deaths
