# Supplementary figures and images for: Modified WCRF/AICR Score and All-Cause, Digestive System, Cardiovascular, Cancer and Other-Cause-Related Mortality: A Competing Risk Analysis of Two Cohort Studies Conducted in Southern Italy
Source: Nutrients. 2021 Nov 10;13(11):4002. doi: 10.3390/nu13114002 (PMC8620807; doi:10.3390/nu13114002)

Supplementary figure S1. NUTRIHEP study Participant: Flow Chart

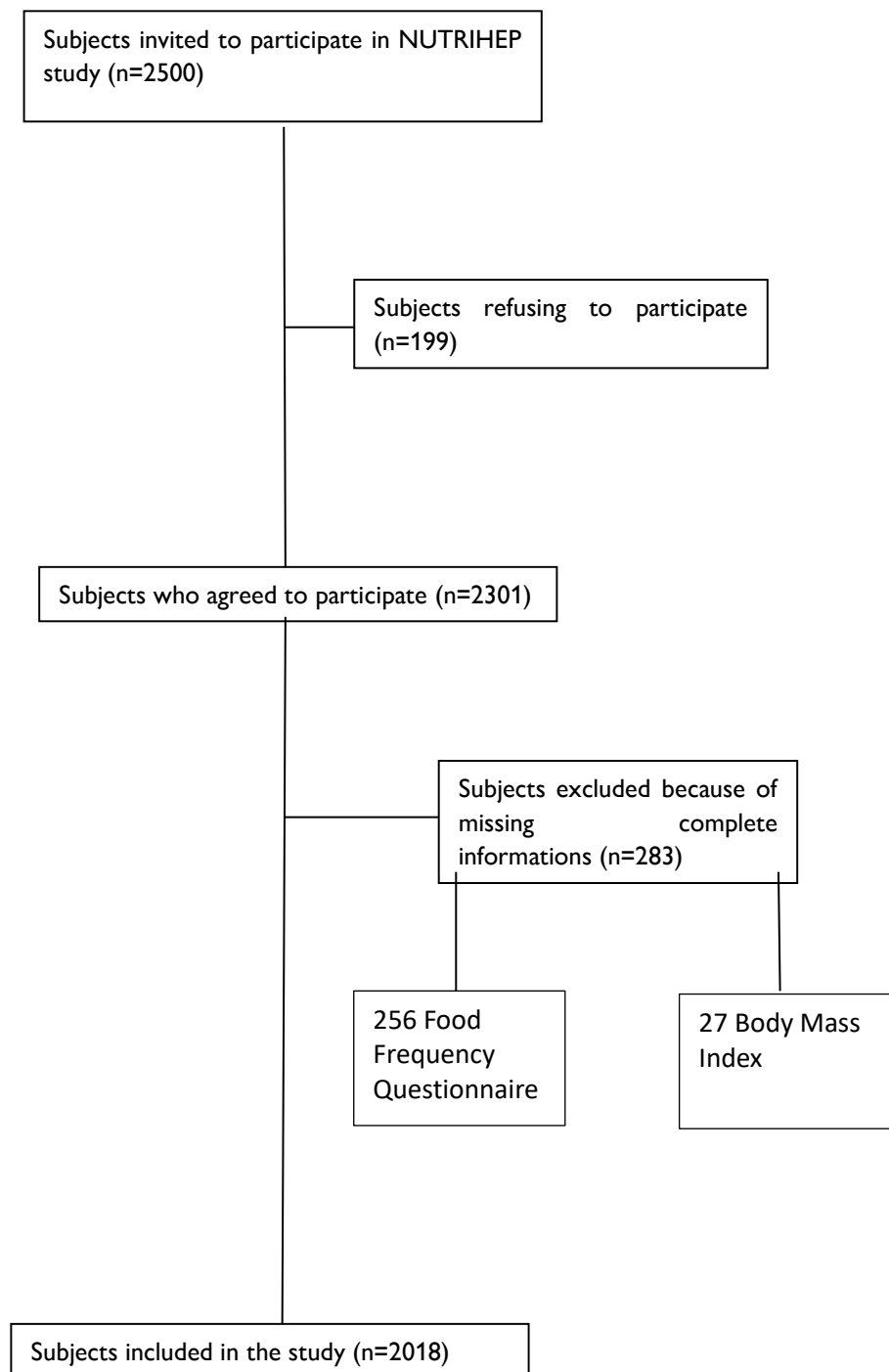

Supplement: Supplementary file 1 [file nutrients-13-04002-s001.zip › supplementary.figure S1.pdf]

Supplementary figure S2. MICOL/PANEL study Participant Flow Chart

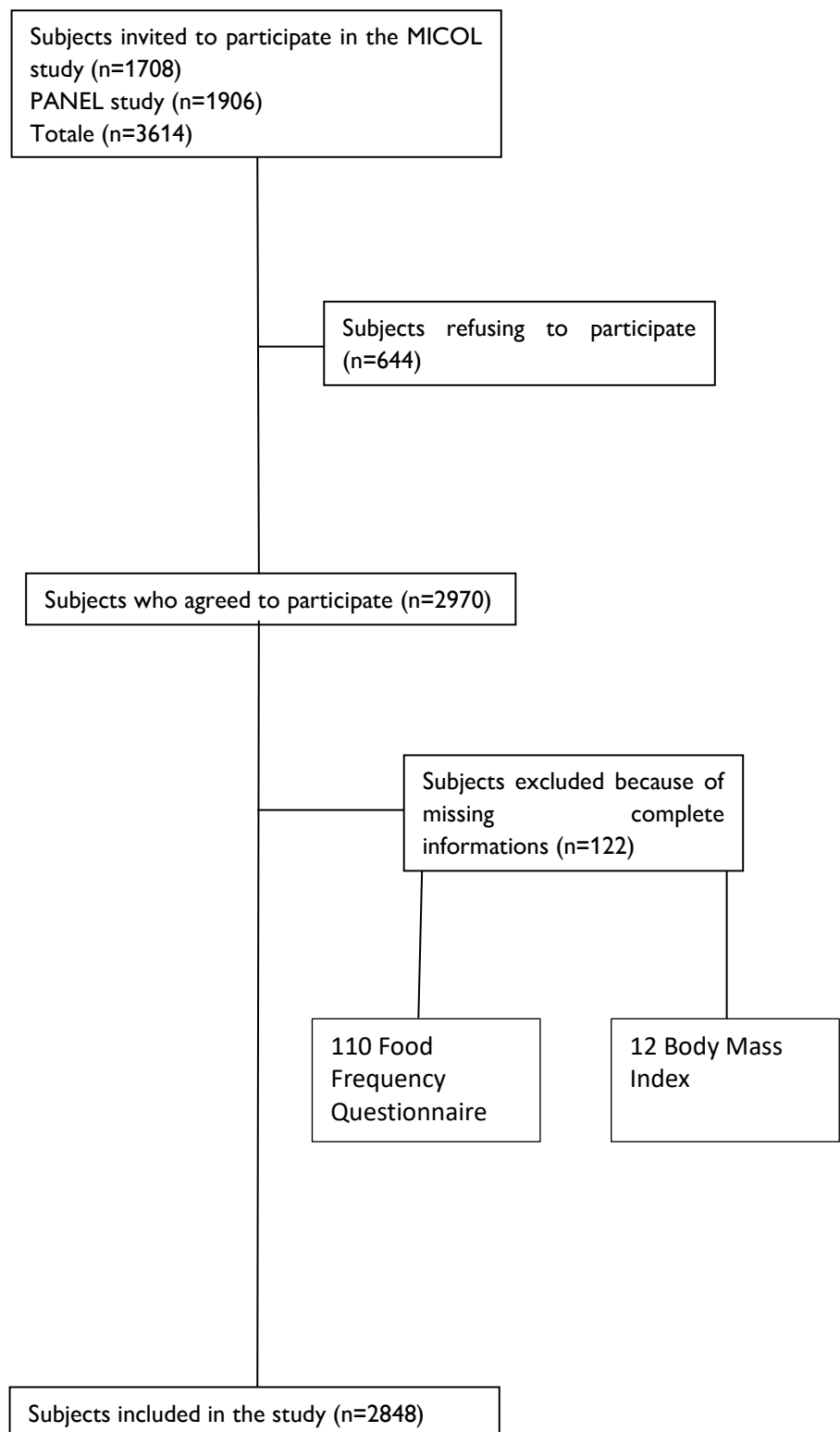

Supplement: Supplementary file 1 [file nutrients-13-04002-s001.zip › supplementary.figure S2.pdf]
